# Effects of microbiota‐based interventions on depression and anxiety in children and adolescents—A systematic review

**DOI:** 10.1002/jpn3.70092

**Published:** 2025-05-26

**Authors:** Jiayu Hu, Yan Zhang, Chuwen Liu, Antigone Gkaravella, Jinyue Yu

**Affiliations:** ^1^ Department of Population, Policy & Practice, Childhood Nutrition Research Group UCL Great Ormond Street Institute of Child Health London UK; ^2^ Microbiota Division, Department of Gastroenterology and Hepatology, The First Medical Center Chinese PLA General Hospital Beijing China; ^3^ Evidence Synthesis Group, Bristol Medical School University of Bristol Bristol UK

**Keywords:** gut microbiome, mental health, psychobiotics, young people

## Abstract

This study aims to systematically review evidence on gut microbiota‐based interventions for reducing depression‐ and anxiety‐like symptoms in children and adolescents with autism spectrum disorder, irritable bowel syndrome, Prader‐Willi syndrome, below‐average literacy skills or anorexia nervosa, where some individuals may exhibit indicators of depression or anxiety. This review includes evaluated evidence from randomized controlled trials (RCTs) involving children and adolescents aged 3–19 years, identified from PsycINFO, Medline (Ovid version), Web of Science, and the reference lists of existing reviews. Risk of bias were assessed using Risk of Bias Tool (RoB 2) in RevMan (version 5.4, Cochrane Collaboration). The results were qualitatively summarized by describing the main findings across the studies. Of the 1561 studies screened, 10 RCTs with 408 participants were included. Three gut microbiota‐based interventions evaluated were probiotics, prebiotics, and dietary supplementation. Probiotics and dietary supplementation were identified as effective on reducing depression and anxiety in three studies; no significant effects were reported in the remaining seven studies. No evidence supported the effectiveness of prebiotics in reducing depression and anxiety in children and adolescents. Four studies presented low risk of bias, while others showed some bias in the randomization process, allocation concealment, selective reporting, and blinding of the outcome assessment. This review highlights the potential of probiotics and dietary supplements in treating depression and anxiety in children and adolescents. However, the current evidence is constrained by inadequate mental health measurements, participant heterogeneity, and small sample sizes in reviewed studies. Further well‐designed studies are needed to confirm their effectiveness.

## Abstract (highlights)

What is Known

- Gut microbiota‐based interventions, including probiotics, prebiotics and dietary changes, are being widely investigated for their potential effects on reducing depression and anxiety symptoms.
- Some randomized controlled trials (RCTs) have shown potential benefits of gut microbiome interventions on mental health in humans.

What is New

- This review highlights evidence suggesting that *Lactobacillus plantarum* PS128, *Bifidobacterium animalis subsp. Lactis strain BPL1* and dietary supplementation with Omega‐3 PUFAs may help reduce depression and anxiety symptoms in children and adolescents, while also identifying areas requiring further investigation.
- Prebiotics, however, showed no significant effect in alleviating these symptoms in young people.

## 1 INTRODUCTION

In recent years, serious mental health issues among young individuals have risen globally, with approximately one in seven children and adolescents aged 10–19 affected by a mental disorder.^1^ Depression and anxiety are the most common, accounting for around 55% of mental health disorders in Europe.^2^ The COVID‐19 pandemic doubled depressive symptoms and increased anxiety among adolescents.^3^, ^4^ Early interventions are crucial, yet less than half of affected youth receive treatment before age 18.^5^ Addressing depression and anxiety in young populations has become a significant public health concern, highlighting the need for further research into effective treatments.

With increased understanding of the gut microbiota's role on children and adolescents' mental health,^6^, ^7^ several preclinical studies have investigated gut microbiota‐based interventions for depression and anxiety, including probiotics, prebiotics, and dietary changes. A summary of these preclinical studies is presented in Supporting Information: Table S1.^8^, ^9^, ^10^, ^11^, ^12^, ^13^, ^14^, ^15^, ^16^, ^17^, ^18^, ^19^, ^20^ Probiotic bacteria such as *Lactobacillus* and *Bifidobacterium* have shown potential in alleviating depression and anxiety symptoms in rodents.^8^, ^9^, ^10^ Prebiotics like fructooligosaccharides (FOS) and galactooligosaccharides (GOS) have also been effective in rodent models.^15^, ^16^ Moreover, certain diets and supplements like magnesium have shown promise in reducing depression and anxiety symptoms.^19^, ^20^ However, a diet enriched with Omega‐3 fatty acids showed no benefits for treating depressive and anxiety symptoms in rodent models.^18^

Apart from those preclinical studies, randomized controlled trial (RCT) have showed the potential effects of gut‐microbiome interventions on human mental health.^21^, ^22^ However, current findings are inconsistent.^23^, ^24^, ^25^ Moreover, no systematic review focused on children and adolescents. Therefore, this systematic review aims to assess the impact of gut microbiota‐based interventions on depressive and anxiety symptoms in children and adolescents aged 3–19 years. By including studies that evaluate these symptoms using validated scales, this review provides a comprehensive analysis of the evidence. Moreover, this review encompasses children with pre‐existing conditions, such as depressive disorder, autism spectrum disorder (ASD), irritable bowel syndrome (IBS), Prader‐Willi syndrome, and anorexia nervosa (AN), where depressive and anxiety symptoms were assessed.

## 2 METHODS

Literature was sourced from: PsycINFO, Medline (Ovid version), Web of Science, and the reference lists of existing reviews. The search terms were grouped into four main themes: (1) Microbiome and the MGB axis; (2) microbiota interventions; (3) depression and anxiety; (4) children and adolescents. Supporting Information: Table S2 provides detailed information on the search terms used. The initial searches were performed on January 1, 2023 and updates on September 28, 2023. This study did not require institutional review board (UCL Research Ethics committee) approval.

Inclusion and exclusion criteria for this study are detailed in Supporting Information: Table S3. Details are included for: Population (P); Intervention (I); Comparisons (C); Outcome (O); Study design (S). Studies were included if they focused on children and adolescents aged 3–19 years, as this age range encompasses both childhood and adolescence, capturing developmental stages where depressive and anxiety symptoms may emerge and be addressed. Participants were required to exhibit depressive or anxiety symptoms, assessed using validated scales such as Child Behavior Checklist (CBCL), Children Mood and Feelings Questionnaire ‐ child short version (SMFQ), and Behavior Assessment System for Children – 2 (BASC‐2), with no recent history of selective serotonin reuptake inhibitor (SSRI) use or engagement in psychotherapy. Eligible interventions were limited to gut microbiota‐based approaches, with comparators including placebo or other non‐gut microbiota‐based interventions. Studies were included regardless of whether depressive or anxiety symptoms were the primary or secondary outcomes, provided these symptoms were assessed. Additional pre‐existing conditions, such as those associated with ASD, anorexia nervosa, irritable bowel syndrome, Prader–Willi syndrome, and below‐average literacy skills were also considered if related to depressive or anxiety symptoms.

Exclusion criteria included studies involving adults, infants, or nonhuman populations (e.g., rodent/animal studies). Interventions unrelated to gut microbiota, such as psychological or pharmacological therapies, SSRIs, or psychotherapy, were excluded. Studies without valid control or comparator groups, as well as those not assessing depressive or anxiety symptoms, were also excluded. Finally, only experimental RCTs were included, while reviews, brief reports, protocols, and observational studies were excluded from the analysis.

Titles and abstracts were screened against the inclusion and exclusion criteria by two independent investigators (J.H and Y.Z) using Rayyan Software. Discrepancies were discussed with a third researcher (J.Y). Data extraction (J.H and C.L) and risk of bias assessment (J.H and Y.Z) were performed by two researchers independently, the general reporting quality of the selected studies was assessed based on the Consolidated Standards of Reporting Trials (CONSORT) 2010 statement.^26^ Risk of bias was evaluated using the Risk of Bias Tool (RoB 2) in RevMan (version 5.4, Cochrane Collaboration). Studies were judged to be at “low” or “high” risk of bias or to raise “some concerns”. Disagreements were resolved by consulting a third investigator (J.Y). An example of the risk of bias assessment using the revised Cochrane risk‐of‐bias tool for randomized trials (RoB 2) is provided in Supporting Information: Appendix S1.

## 3 RESULTS

**Figure 1:**
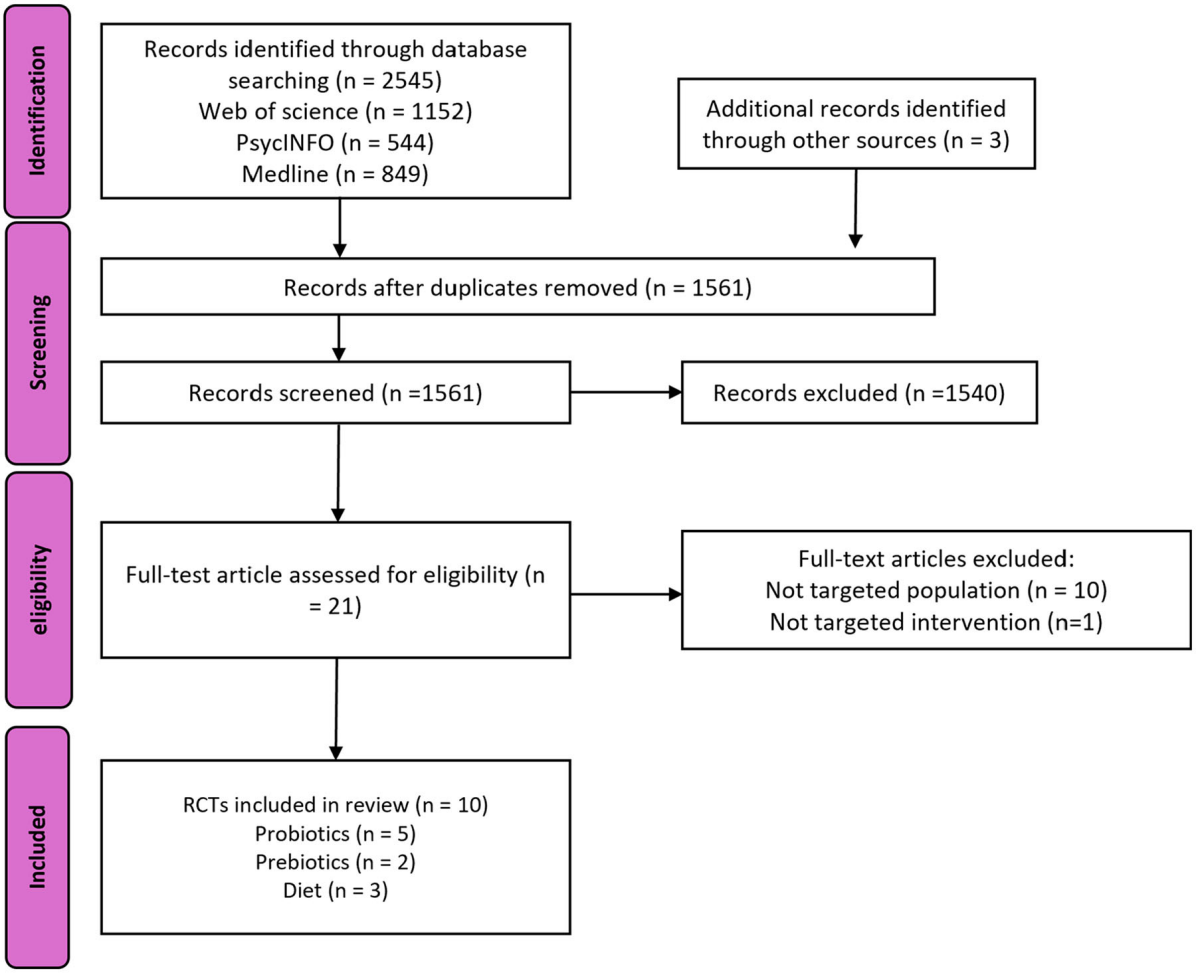
PRISMA flow chart. PRISMA, Preferred Reporting Items for Systematic reviews and Meta‐Analyses.

The literature search across PsycINFO, Medline, and Web of Science yielded 1561 records. With three additional records were identified through the reference lists of other reviews. After deduplication and screening by title and abstract, 21 records remained (see PRISMA flow chart, Figure 1). Ultimately, 10 studies were selected for this review. Exclusions during the full‐text screening stage were due to incorrect target population (*n* = 10). A summary of study characteristics is presented in Table 1.^27^, ^28^, ^29^, ^30^, ^31^, ^32^, ^33^, ^34^, ^35^, ^36^, ^37^ General reporting quality was listed in Table 2.

**Table 1:** Characteristics of the included studies.

Authors (year)	Sample		Methods		Outcome measures			Results	
	Number of participants (*N*)	Participants	Intervention groups	Control groups	Measures of depression	Measures of anxiety	Measures of gut microbiota	Mental health outcomes	Gut microbiota outcomes
*Probiotic studies*									
Eugene Arnold et al., 2019^27^	10	3–12 years old children with ASD, anxiety, and GI symptoms	Probiotic mix (VISBIOME), containing eight probiotic species, mostly *Lactobacillus* and *Bifidobacterium* (*n* = 6)	Placebo (*n* = 4)	/	Parent‐rated anxiety scale for ASD (PRAS‐ASD), Social Responsiveness Scale (SRS), Children's Sleep Habits Questionnaire (CSHQ)	16S rRNA V4 sequencing	No statistically significant differences between the probiotic and placebo group	Abundance of *Lactobacillus* correlated significantly with the PedsQL score
Santocchi et al., 2020^28^	63	Preschoolers aged 18–72 months with ASD, mean age 4.2	Probiotics (De Simone Formulation) (*n* = 31)	Placebo (*n* = 32)	Child Behavior Check List 1,5‐5 (CBCL 1,5‐5)	Child Behavior Check List 1,5‐5 (CBCL 1,5‐5)	/	The change of CBCL total scores was smaller in the Probiotics group than the Placebo group from T0–T2, which means probiotics did not have positive impacts on reducing depression and anxiety	/
Parracho et al., 2010^29^	17	4–16 years old children with diagnosis of ASD	*Lactobacillus plantarum* WCFS1 (*n* = 17)	Placebo (*n* = 17)	/	Development Behaviour Checklist – Primary carer version (DBC‐P), Total behaviour Problem score (TBPS) – anxiety problem	Bacterial population levels were examined using fluorescence in situ hybridization (FISH)	No significant difference between the probiotic and placebo group regarding the anxiety scores	No statistically significant sequence effect was observed, probiotic supplementation resulted in significantly higher Lab158 counts, and significantly lower Erec482 counts, compared to placebo
Liu et al., 2019^30^	71	7–15 years old boys with ASD in Taiwan	PS128 (*n* = 36)	Placebo (*n* = 35)	/	CBCL ‐ Anxiety	/	Scores of CBCL‐Anxiety nominally reduced in the PS128 group (*p* = 0.02)	/
Amat‐Bou et al., 2020^31^	39	Children 2‐19 years old with Prader‐Willi syndrome (PWS) Mean age 10.4	*Bifidobacterium animalis* subsp. Lactis strain BPL1 (*n* = 35)	Placebo (*n* = 36)	Parent‐ report form CBCL	Parent‐ report form CBCL	QIAamp PowerFecal DNA kit	BPL1 induced modest but significant improvements in withdrawn/depression symptoms, B = −4.7, 95% CI [−0.9, −0.3], *p* = 0.037.	Microbiota composition was not substantially modified by probiotic treatment
*Prebiotic*									
Capitão et al., 2020^32^	35	7–9 years old children with low reading scores 24 female and 11 males	B‐GOS (*n* = 17)	Placebo (maltodextrin) (*n* = 18)	SMFQ	STAIC SMFQ	/	no significant changes after the prebiotic treatment	/
Grimaldi et al., 2018^33^	30	4–11 years old children with ASD Gender not reported	B‐GOS (*n* = 13)	Placebo (*n* = 13)	/	SCAS‐P	16S rRNA gene amplification via next‐generation sequencing (NGS) and bioinformatics analysis	Not significantly affected, but the restricted diet can have impacts on the social skills between placebo group and B‐GOS group	Significant increase of *Bifidobacterium* spp., *Ruminococcus* spp., *Lachnospiraceae* family, *Eubacterium dolchum*, TM7‐3 family and Mogibacteria
*Diets*									
Shulman et al., 2017^34^	84	Children 7–18 years of age meeting pediatric Rome III IBS criteria	Fiber used was psyllium (*n* = 37)	Placebo used was maltodextrin (*n* = 47)	Behavior Assessment System for Children – 2 (BASC‐2)	Behavior Assessment System for Children – 2 (BASC‐2)/	16S rRNA (V3‐V5) on 454 platform, and QIIME was used for bioinformatic analysis	Scores for depression and anxiety were in the normal range, no significant difference between fiber and placebo group	At baseline, enriched in Bacteroidetes and decreased Firmicutes in fiber group at phylum level. Class level, increased in Bacteroidia, decreased in Clostridia.
Trebatická et al., 2017^35^	35	11–17 years old patients with depressive disorder 8 males and 27 females	Omega‐3 FA (*n* = 17) 12 weeks	Omega‐6 FA (*n* = 18)	CDI	/	/	Significant reductions in CDI scores in patients received 12 weeks Omega‐3 (*p* = 0.034) CDI: Omega‐3 < Omega‐6	/
Manos et al., 2018^36^	24	Adolescent females with restrictive anorexia nervosa and anxiety trait	Four daily PUFAs capsules (*n* = 12)	Placebo capsules (*n* = 12)	CES‐D	BAIT	/	No difference between omega‐3 PUFA and placebo groups	/

Abbreviations: ASD, autism spectrum disorder; B‐GOS, bimuno galactooligosaccharide; BASC‐2, Behavior Assessment System for Children ‐ 2; BAIT, Beck Anxiety Inventory‐Trait; CBCL, Child Behaviour Checklist; CDI, Children's Depression Inventory; CES‐D, Center for Epidemiologic Studies Depression Scale; CSHQ, Children's Sleep Habits Questionnaire; DBC‐P, Development Behaviour Checklist ‐ Primary carer version; FA, fatty acids; PRAS‐ASD, parent‐rated anxiety scale for ASD; PS128, *Lactobacillus plantarum* PS128; PUFAs, polyunsaturated fatty acids; SCAS‐P, Spence's Children Anxiety Scale‐Parent version; SMFQ, Children Mood and Feelings Questionnaire ‐ child short version; SRS, Social Responsiveness Scale; STAIC, State‐Trait Anxiety Inventory for children; TBPS, Total Behaviour Problem score.

**Table 2:** General reporting quality of included studies.

Section	Description	Amat‐Bou et al.^31^	Eugene Arnold et al.^27^	Capitão et al. 32	Grimaldi et al.^33^	Liu et al.^30^	Manos et al.^36^	Parracho et al.^29^	Santocchi et al.^28^	Shulman et al.^34^	Trebatická et al.^35^
Title and Abstract	**Title:** Identify the study as a randomized trial in the title.	**Y**	N	Y	N	Y	Y	N	Y	Y	N
	**Abstract**: Provide a structured summary of trial design, methods, results, and conclusions.	Y	Y	N	Y	Y	Y	Y	Y	Y	Y
Introduction	**Background and objectives:** Explain the scientific background and rationale for the trial, and state the specific objectives or hypotheses.	Y	Y	Y	Y	Y	Y	Y	Y	Y	Y
Methods	**Trial design:** Describe the trial design (such as parallel, factorial) and include details of any changes to methods after trial commencement.	Y	Y	Y	Y	Y	Y	Y	Y	Y	Y
	**Participants:** Include eligibility criteria for participants and settings where the data were collected.	Y	Y	Y	Y	Y	Y	Y	Y	Y	Y
	**Interventions:** Provide detailed descriptions of the interventions for each group and how and when they were administered.	Y	Y	Y	Y	Y	Y	Y	Y	Y	Y
	**Outcomes:** Define primary and secondary outcome measures, and, when applicable, any methods used to enhance the quality of measurements.	Y	Y	Y	Y	Y	Y	Y	Y	Y	Y
	**Sample size:** Explain how the sample size was determined, including any interim analyses and stopping guidelines.	N	Y	N	Y	Y	N	Y	N	N	Y
	**Randomization: Sequence generation**: Describe the method used to generate the random allocation sequence, including details of any restrictions.	Y	N	N	N	Y	Y	N	Y	Y	Y
	**Randomization: Allocation concealment:** Describe the method used to implement the random allocation sequence.	Y	N	N	N	Y	Y	N	Y	Y	Y
	**Randomization: Implementation:** Who generated the allocation sequence, who enrolled participants, and who assigned participants to interventions.	Y	N	N	N	Y	Y	N	Y	Y	Y
	**Blinding**: Describe any measures used to blind participants, personnel, and outcome assessors from knowing the intervention groups. Provide any information about the resemblance of interventions.	Y	N	N	N	Y	Y	N	Y	Y	Y
	**Statistical methods**: Describe all statistical methods used to compare groups for primary and secondary outcomes, including methods for additional analyses, such as subgroup analyses and adjusted analyses.	Y	Y	Y	Y	Y	Y	Y	Y	Y	Y
Results	**Participant flow**: Provide a diagram of the flow of participants through each stage of the trial (enrollment, intervention allocation, follow‐up, and data analysis).	Y	N	N	Y	Y	Y	N	Y	Y	Y
	**Recruitment**: Dates defining the periods of recruitment and follow‐up.	Y	Y	Y	Y	Y	Y	Y	Y	Y	Y
	**Baseline data**: Provide baseline demographic and clinical characteristics of each group.	Y	Y	Y	N	Y	Y	N	Y	Y	N
	**Numbers analyzed**: Number of participants (denominator) in each group included in each analysis and whether the analysis was by the original assigned groups.	Y	Y	Y	Y	Y	Y	Y	Y	Y	Y
	**Outcomes and estimation**: For each primary and secondary outcome, present the results for each group, and the estimated effect size and its precision (e.g., 95% confidence interval).	Y	Y	Y	Y	Y	Y	Y	Y	Y	Y
	**Ancillary analyses**: Results of any other analyses performed, including subgroup analyses and adjusted analyses, distinguishing prespecified from exploratory.	Y	Y	Y	Y	Y	Y	Y	Y	Y	Y
	**Harms**: Important adverse events or side effects in each intervention group.	N	Y	Y	N	N	Y	N	N	N	Y
Discussion	**Limitations**: Discuss trial limitations, addressing sources of potential bias, imprecision, and multiplicity of analyses.	Y	Y	Y	Y	Y	Y	Y	Y	Y	Y
	**Generalizability**: Discuss the generalizability (external validity) of the trial findings.	N	Y	N	N	N	Y	N	N	N	N
	**Interpretation**: Interpret results in the context of current evidence.	Y	Y	Y	Y	Y	Y	Y	Y	Y	Y
Other Information	**Registration**: Registration number and name of the trial registry.	Y	Y	Y	Y	Y	Y	Y	Y	Y	Y
	**Protocol**: Where the full trial protocol can be accessed, if available.	Y	Y	Y	Y	Y	Y	Y	Y	Y	Y
	**Funding**: Sources of funding and other support (such as supply of drugs), role of funders.	Y	Y	Y	Y	Y	Y	Y	Y	Y	Y

### 3.1 Effects of probiotic related intervention

In total, five studies on probiotic interventions involved 200 participants, ranging in age from 2 to 19 years old. Four studies reported gender: 37 female and 142 males.

Amat‐Bou et al.^31^ reported that a 12‐week consumption of *Bifidobacterium animalis* subsp. *lactis* strain BPL1 had modest positive impact on withdrawn/depression symptoms, with *B* = −4.7, 95% CI [−0.9, −0.3], *p* = 0.037, though the microbiota composition was not substantially changed by probiotic treatment. Another study examined the influence of a 4‐week consumption of probiotic *Lactobacillus plantarum* PS128 on boys diagnosed with ASD.^30^ The results highlighted a significant reduction (*p* = 0.02) in Child Behaviour Checklist (CBCL)‐anxiety scores within the PS128 group, underlining its potential benefits in alleviating anxiety‐like symptoms.

Conversely, a study^28^ on De Simone Formulation (DSF) probiotics in pre‐schoolers with autism showed that the change of CBCL total scores was smaller in the probiotic group than the placebo group, suggesting that the probiotics were not beneficial on reducing depression and anxiety symptoms. Similarly, another study^29^ investigated the effects of a 12‐week‐intervention using *L. plantarum* WCFS1 on children with ASD showed no significant difference between the probiotic and placebo group regarding the anxiety scores. Finally, a study^27^ investigated the effects of a 19‐week‐intervention using a probiotic mix named VISBIOME explored its impacts on the quality of life among children with ASD. This probiotic mix contained four strains of *Lactobacillus*, three strains of *Bifidobacteria*, one strain of *S. thermophiles*, and starch. Results of this study revealed an increase of *Lactobacillus* post‐intervention. However, the intervention did not produce a significant impact on the Parent‐Rated Anxiety Scale for Autism Spectrum Disorder (PRAS‐ASD) scores in comparison to a placebo group, suggesting no substantial reduction in anxiety symptoms.

### 3.2 Effects of prebiotic related intervention

In total, two studies involving 65 participants aged 4–11 years old were included. One study did not specify gender,^33^ the other study consisted of 35 participants (24 females, 11 males).^32^ The average age of the participants ranged from 7.7 to 8.84 years. Both studies involved a diverse clinical population, including children and adolescents affected by ASD and reading problems.^32^, ^33^

Neither study showed significant effects.^32^, ^33^ Capitão et al.^32^ explored the impact of B‐GOS supplementation on children's reading and cognitive abilities and measured the influence of a 12‐week consumption of B‐GOS on anxiety. The findings showed no significant alterations in State‐Trait Anxiety Inventory (STAIC) or Short Mood and Feelings Questionnaire (SMFQ) scores post‐prebiotic treatment, indicating that B‐GOS did not markedly affect children's anxiety symptoms.

Another study^33^ assessed the effects of exclusion diets combined with a 6‐week B‐GOS prebiotic intervention in children with ASD. This study also found no notable shifts in Spence Children's Anxiety Scale – Parent (SCAS‐P) scores, despite a rise in the *Lachnospiraceae* bacteria. Prebiotic intervention did not display significant effects on reducing depression and anxiety symptoms in the included young population.

### 3.3 Effects of diets related intervention

The studies involved 143 participants, aged 7–21, with a majority being female (*n* = 99), and 44 males. The average age of participants ranged from 13.1 to 15.6 years. The studies included children and adolescents with IBS, depressive disorders, restrictive AN, and anxiety trait.

One study^34^ assessed a 6‐week psyllium fiber intervention on the abdominal pain and stool patterns in children with IBS, there was no significant difference between fiber and placebo group in terms of depression and anxiety scores.

Diets enriched with Omega‐3 PUFAs were also examined. One study^35^ explored the effects of a 12‐week intervention with Omega‐3 PUFAs in treating depressive symptoms in children and adolescents diagnosed with depressive disorder (DD) and mixed anxiety depressive disorder (MADD). The findings revealed that Omega‐3 PUFAs substantially reduced Children's Depression Inventory (CDI) scores (*p* = 0.034), highlighting its positive impacts on alleviating depression symptoms in children and adolescents.

However, another study^36^ evaluating the effectiveness of a 12‐week Omega‐3 PUFAs supplementation for treating anxiety traits in adolescents females diagnosed with restrictive AN found that the Beck Anxiety Inventory (BAIT) scores and the Center for Epidemiologic Studies Depression Scale (CES‐D) scores decreased in both the Omega‐3 PUFAs and placebo group. Thus, Omega‐3 PUFAs did not exhibit any superior efficacy compared to the placebo. Overall, while Omega‐3 PUFAs demonstrate potential benefits in reducing depression and anxiety symptoms in children and adolescents, similar reduction was noted in the placebo group.

### 3.4 Risk of bias assessment

#### 3.4.1 Probiotics studies

**Figure 2:**
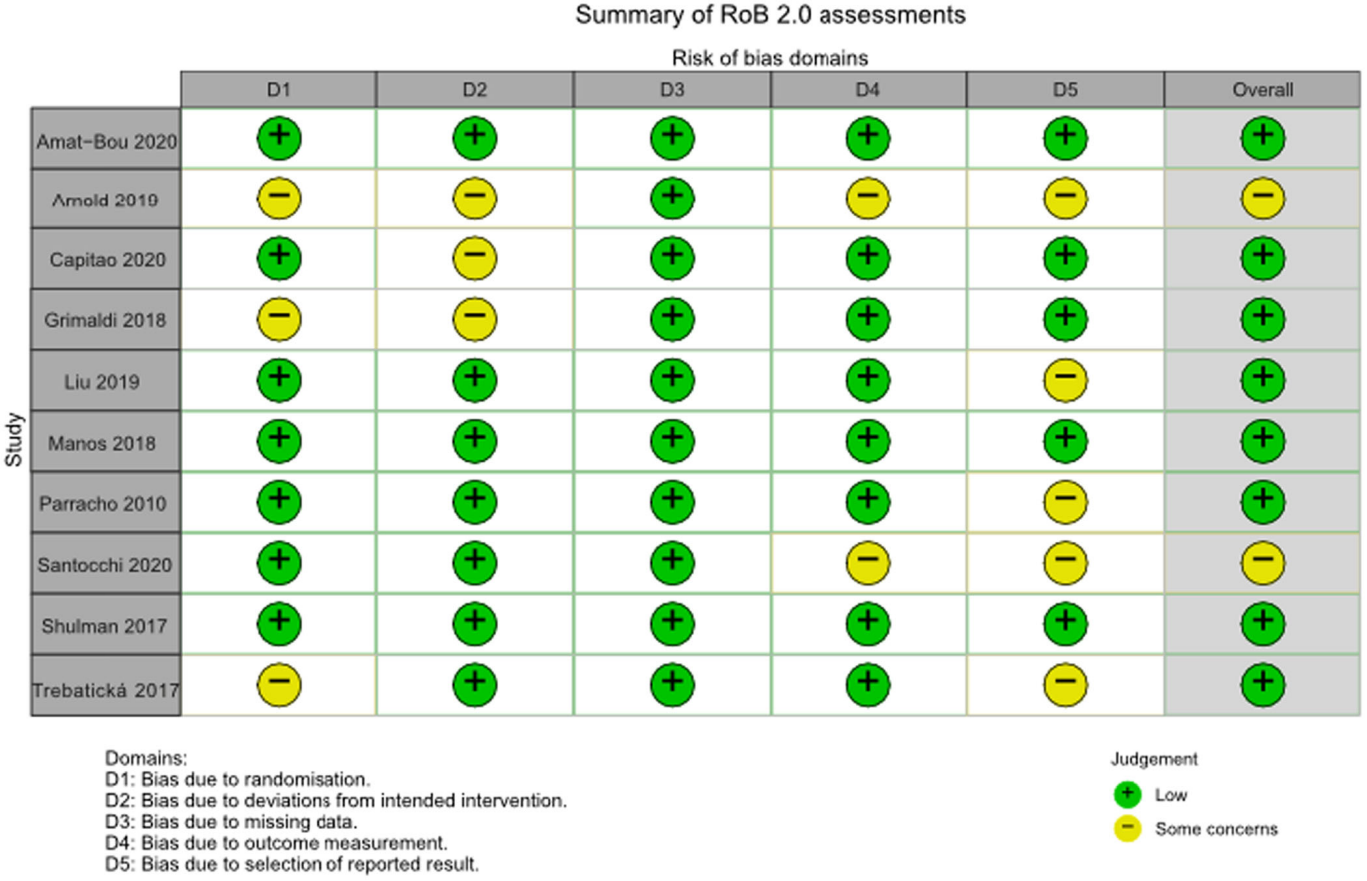
Risk of bias of the included studies.

Of the five studies focused on probiotic interventions, three demonstrated a low risk of bias.^29^, ^30^, ^31^ One raised concerns about bias concerning randomization allocation, allocation concealment, and the blinding of participants and personnel^27^ (Figure 2). While described as a randomized crossover feasibility pilot trial, this study lacked details about randomization methods. The authors acknowledged that participants might have accurately guessed their treatment assignments, casting doubt on the effectiveness of blinding; additionally, this study did not address allocation concealment.^27^ In terms of selective reporting, four studies raised up some problems as there was insufficient information on whether data were analyzed according to a pre‐specified analysis plan.^27^, ^28^, ^29^, ^30^ Moreover, two studies might contain detection bias due to unclear information on whether the assessors were aware of the intervention received by study participants or not.^27^, ^28^ These biases can have impacts on the studies' internal validity and the reliability of their reported findings. Future research should mitigate these issues by implementing a more rigorous randomization method, detailed reporting of blinding process, and a more explicit allocation concealment.

#### 3.4.2 Prebiotics studies

One study identified concerns regarding allocation concealment, the blinding of participants, and other potential biases.^32^ Despite being labeled as a double‐blind study, the methodology lacked clarity on how blinding was implemented and whether group allocation remained concealed from participants until their enrollment and assignment to interventions.^32^ Furthermore, the study's use of self‐reported mood assessments and food diaries might introduce additional biases. Another study also faced ambiguity in its blinding procedure.^33^ Although characterized as a double‐blind study, it remains uncertain whether participants were informed about their group assignments. To address these issues, future research should add more information on the blinding process and consider using objective assessments to reduce bias.

#### 3.4.3 Dietary supplementation studies

Two studies presented low risk of bias,^34^, ^36^ while another showed potential bias issues in random sequence generation and selective reporting^35^ (Figure 2). The study by Trebatická et al.^35^ neither provided details on allocation concealment nor clarified whether participants or intervention deliverers were aware of assigned treatments during the research. Moreover, the study lacked information on whether the data was analyzed according to the pre‐specified analysis plan. There was no multiple eligible outcome measurements and analyses of the data within the outcome domain. To improve methodological rigor, future research should prioritize pre‐specified analysis plans and ensure that outcome measurements and analyses are consistent with these plans. Researchers should also explicitly illustrate the allocation concealment and blinding process to mitigate bias.

## 4 DISCUSSION

This review synthesizes findings from 10 RCTs, analysing three types of gut microbiota‐based interventions for treating depression and anxiety in children and adolescents. The results were mixed, though probiotic and diet supplements showed some potential benefits in treating depression and anxiety among children and adolescents. Potential effective dose, duration, and frequency (e.g., ${5\times 10}^{8}$ CFU/d or ${3\times 10}^{10}$ CFU/d for probiotics) for treating depression and anxiety in clinical contexts^30^, ^35^ were identified. However, given the significant participant heterogeneity, clinicians should interpret these results cautiously, as effectiveness might vary among specific populations.

Specific probiotics, such as *L. plantarum* PS128 and *B. animalis subsp. Lactis strain BPL1*, demonstrated beneficial effects in reducing depression and anxiety symptoms in children and adolescents.^30^, ^31^ Conversely, other probiotics like probiotic mix (VISBIOME), *L. plantarum WCFS1*, and Probiotics with DSF, had no significant impacts. Furthermore, the probiotic mix VISBIOME did not affect anxiety levels in children and adolescents.^27^ For prebiotic interventions, B‐GOS was ineffective in treating depression or anxiety.^32^, ^33^ Dietary interventions, particularly supplementation with Omega‐3 PUFAs, demonstrated potential benefit for decreasing depression and anxiety symptoms, but similar reductions were sometimes observed in the placebo group.^35^, ^36^ Supplementation with psyllium fiber did not reduce depression and anxiety symptoms either.^34^

While probiotic and dietary interventions demonstrated statically significant effects, their clinical effectiveness remains uncertain due to several limitations that existed in reviewed studies. First, only one study examined these symptoms as primary outcome,^35^, ^37^ while others examined anxiety comorbid with AN or primarily evaluated other outcomes such as Gastrointestinal symptoms, cognition, IBS, ASD symptoms, and Prader–Willi Syndrome, with depression and anxiety symptoms measured as co‐occurring issues.^27^, ^28^, ^29^, ^30^, ^31^, ^32^, ^33^, ^34^, ^36^

These studies reliance on singular mental health measures reduces reliability.^27^, ^35^ Measurement inconsistencies further hinder synthesis across studies. For example, the CDI focuses on child‐specific symptoms, while CES‐D assesses depressive symptoms across age groups.^38^ Self‐reported scales, again the CDI, CES‐D, and also BAIT^35^, ^36^ might introduce bias, while parent‐reported measures, like the Development Behaviour Checklist (DBC)^38^– primary carer version.^29^, ^31^, ^33^ could reflect parental expectations and affect the results. Combining self‐ and parent‐reported measures could improve reliability.

Second, participant heterogeneity was high, as we included studies with children without clinical diagnoses of depression or anxiety. We acknowledged that this diversity complicates conclusions on intervention effectiveness. Third, small sample sizes limited study power and statistical significance.^27^, ^31^, ^33^, ^35^, ^36^, ^37^ Additionally, short intervention durations in some studies^29^, ^30^, ^31^ (e.g., 12 weeks for scale assessing 24–24 changes) in Amat‐Bou et al.^31^ hampered meaningful outcomes. Few studies assessed long‐term or posttreatment effects, with only one examining sustained impacts.^35^

Finally, some studies exclusively recruited female or male participants,^30^, ^36^ reducing generalizability. Future research should prioritize rigorous multi‐center RCT with larger, diverse samples and standardized measures across genders. Longer durations with follow‐ups are crucial for understanding lasting effects. Incorporating gut microbiota analyses could illuminate mechanisms underpinning interventions. Rigorous study designed with randomization allocation concealment and blinding are essential, along with analyses of participant dropout characteristics. Moreover, to validate the clinical efficacy of probiotics, prebiotics, dietary interventions, future studies should also focus on clinical populations, as evidence suggests probiotics effects diminish in nonclinical groups.^30^ Investigating psychiatric samples will clarify the therapeutic potential of gut microbiota‐based interventions.

This is the first systematic review to summarize and analyse gut microbiota‐based interventions for treating depression and anxiety in children and adolescents. It highlighted critical gaps and implications for future clinical practice and research. The studies incorporated in this review were all RCTs, offering relatively more reliable evidence on the interventions' effectiveness. As such, this review lends more weight to drawing a causal relationship between gut microbiota‐based interventions and mental health outcomes in children and adolescents.

However, this review also has some limitations. First, the limited number of studies included in this review could be inadequate to derive a robust conclusion about the effectiveness of each intervention. The actual effects of the interventions may be over‐ or underestimated. However, the 10 RCTs in this review could serve as a starting point for future research by demonstrating the potential effects of wide assortment of the microbiome related treatment. Future systematic reviews on both experimental and observational studies in this area are worth to be conducted. Secondly, gray literature was not considered in this review to ensure methodological quality, elevating the risk of publication bias. Gray literature can offer insights not found within commercially published works and might encompass studies with null or negative results.^39^ Moreover, while this review focused on gut microbiota‐targeted interventions, physical exercise emerges as a promising alternative for future exploration in this field. Although exercise is not a direct gut microbiota‐targeted intervention, one study^37^ found that regular aerobic exercise increased certain beneficial gut bacteria, such as *Coprococcus* and *Ruminococcus obeum*. These microbiota changes coincided with significant improvements in depressive symptoms measured by the Chinese version of Patient Depression Questionnaire‐9 (C‐PHQ‐9) scale. This suggests a potential indirect mechanism through which exercise could influence mental health by modulating gut microbiota. Future studies should integrate exercise as a complementary or alternative approach in gut microbiota research, particularly to examine its interaction with other interventions such as probiotics or diet. Most of the participants included in the current review were neurodivergent individuals with ASD and PWS. Only four studies focused on neurotypical children and adolescents with conditions such as depressive disorder, anorexia nervosa, IBS, or below‐average literacy skills. To better understand the effect of microbiota modulation across diverse populations, further research on neurotypical individuals is necessary. Additionally, future studies should also compare the mechanistic differences in microbiota modulation between neurotypical and neurodivergent individuals to provide deeper insights for this type of intervention.

Moreover, while this review includes and discusses diet‐related interventions, focusing solely on their effects on the microbiome may overlook the broader mechanisms through which diet influences mental health. It should be noticed that beyond its direct impact on the microbiota, diet shapes metabolic pathways, inflammatory responses, and immune functions. Future research should examine the multifaceted ways in which diet interacts with microbiota and other physiological systems. By integrating these dimensions, we can better understand its therapeutic potential for mental health problems.

## 5 CONCLUSION

This review offers insights into the therapeutic effects of gut microbiota‐based interventions for the treatment of depression and anxiety in children and adolescents. Although some studies showed initial effectiveness, further exploration of clinical efficacy is needed. Future studies should prioritize well‐designed, multi‐center RCTs with larger, more diverse samples to improve generalizability, as well as standardized outcome measures assessing depressive and anxiety symptoms to enhance comparability. Longitudinal studies with follow‐up assessments are needed to evaluate the sustainability of intervention effects. Pre‐registration of analysis plans and age‐stratified analyses can further enhance the rigor and relevance of future research. Additionally, incorporating gut microbiota composition analyses could provide mechanistic insights, supporting the development of precision‐targeted therapies.

## CONFLICT OF INTEREST STATEMENT

The authors declare no conflicts of interest.

## Supporting information

Supporting information.
