# Supplementary material for: Effects of microbiota‐based interventions on depression and anxiety in children and adolescents—A systematic review
Source: J Pediatr Gastroenterol Nutr. 2025 May 26;81(2):404–16. doi: 10.1002/jpn3.70092 (PMC12314587; doi:10.1002/jpn3.70092)
Supplement: Supplementary file 1 — Supporting information. [file JPN3-81-404-s001.docx]

**Online-Only Supplemental Material**

**Effects of microbiota-based interventions on depression and anxiety in children and adolescents – a systematic review**

Jiayu Hu,^1^ Yan Zhang,^2^ Chuwen Liu,^1^ Antigone Gkaravella,^1^ Jinyue Yu^1,3^

1. Department of Great Ormond Street Institute of Child Health, University College London, London, UK.
2. Microbiota Division, Department of Gastroenterology and Hepatology, The First Medical Center, Chinese PLA General Hospital, Beijing, China
3. Evidence Synthesis Group, Bristol Medical School, University of Bristol, Bristol, UK.

Table S1 **Summary of Preclinical Evidence on Gut Microbiota-based Intervention**

| Authors (year) | Sample |  | | Methods | Outcome measures | |  |  | | Results |  |  |  |  |
| --- | --- | --- | --- | --- | --- | --- | --- | --- | --- | --- | --- | --- | --- | --- |
|  | **Sample size (N)** | **Animals** | | **Interventions** | **Depressive Behaviors assessments** | | **Anxious behaviors assessments** | **Gut microbiota analysis** | | **Behavioural outcomes** | **Gut microbiota composition outcomes** |  |  |  |
|  | | |  | | | **Probiotics** | | |  | | | |  | **Probiotics** |
| Desbonnet et al., 2010^1^ | 33 | Pregnant Sprague-Dawley rats and their male offsprings | | MS (n = 7); MS + Citalopram (n = 7); MS + *Bifidobacterium infantis* (n = 8) | FST | | / | / | | Chronic treatment with *Bifidobacteria* reduced the immobility time and increased the time spent swimming. | / |  |  |  |
| Karen et al., 2021^2^ | 24 | Pregnant Wistar Rats and their pups | | *Lactobacillus paracasei* | TST | | OFT | 16S rRNA Gene Sequencing | | OFT: spent more time in the central square  TST: significant difference between experimental groups | Early life SSE resulted in a reduction of five anticipated neuroactive microbial metabolites. |  |  |  |
| Kosuge et al., 2021^3^ | / | 7 weeks old Male C57BL/6J and ICR mice | | Heat-sterilized *Bifidobacterium breve* | SIT | | / | 16S rRNA Gene Sequencing | | significantly counteract the impact of CSDS on the duration spent in both the interaction and corner zones | The levels of the *Bifidobacterium* class rose, while those of the *Bacteroidia* class diminished |  |  |  |
| Liao et al., 2019^4^ | 12 litters of mice | Pragnant mice and their litters | | live cells of *Lactobacillus paracasei* PS23; heat-killed cells of *Lactobacillus paracasei* PS23 | FST | | OFT; EPM | / | | FST: immobile time reduced after PS23 supplementation; OFT: higher number of entries to the central zones and increased time spent in the central zone after PS23 supplementation; EPM: no significant differences | / |  |  |  |
| Liu et al., 2016^5^ | 32 | Timed pregnant female and their male pups | | *Lactobacillus plantarum* PS128 | SPT; FST | | OFT; EPM | / | | SPT: PS128 increased sucrose preference; FST: PS128 decreased immobility duration; OFT: PS128 increased Total distance, though there was no change in the time spent in the center; EPM: PS128 decreased the duration in the closed arm while prolonging the time in the open arm. | / |  |  |  |
| Maehata et al., 2019^6^ | 48 | 7 weeks old male C57BL/6J (B6) mice | | Heat-killed *Lactobacillus helveticus* strain MCC1848 | SPT; TST; FST | | NBT; SIT | 16S rRNA genes sequencing | | SPT: the MCC1848 restored the ratio to a similar level to the controls; TST: no difference; FST: no difference; NBT: no difference; SIT: MCC1848 mitigated the decrease in SIT scores and enhanced resilience | The predominant bacterial phylum was *Firmicutes*, succeeded by *Bacteroidetes* and then *Proteobacteria*. |  |  |  |
| Wei et al., 2019^7^ | 40 | 6-8 weeks male C57BL/6J (B6) mice | | Live and heat-killed *Lactobacillus paracasei* PS23 | FST; SPT | | OFT | / | | FST: Both H-PS23 and fluoxetine decreased the duration of immobility, while L-PS23 treatment showed no effect; SPT: L-PS23 and H-PS23 treatments had mild impacts on sucrose preference in mice; OFT: Both PS23 and L-PS23 counteracted the decreased total distance traveled by mice. | / |  |  |  |
|  | | |  | | | **Prebiotics** | | |  | | | |  | **Prebiotics** |
| Burokas et al., 2017^8^ | 69 | 7 weeks old male C57BL/6J mice | | FOS; GOS | Female urine sniffing test; TST; FST | | OFT; defensive marble burying test; EPM, stress-induced hyperthermia | 16S Compositional Analysis of cecal microbiota | | TST: FOS+GOS decreased immobility time; FST: FOS, GOS, and FOS+GOS decreased immobility time; Female urine sniffing test: No significant effect of prebiotic administration on anhedonia; OFT: FOS+GOS treatment increased time in the center; EPM: prebiotic administration had no effects on time spent in open arms; Marble burying: prebiotic treatment reduced the number of buried marbles | The decrease in relative abundance of *Bifidobacterium* |  |  |  |
| Chi et al., 2020^9^ | 62 | 6 weeks old male Sprague-Dawley (SD) rats | | FOS | SPT | | OFT | 16S rRNA sequencing | | SPT: FOS treatment recovered the sucrose intake; OFT: FOS treatment increased the total distance travelled and lowered immobility time | FOS enriched OTUs diversity, affected the *Firmicutes/Bacteroidetes* ratio |  |  |  |
| Jiang et al., 2022^10^ | / | 6-8 weeks old female and male C57BL/J mice | | FOS; GOS | FST | | OFT | 16S rRNA sequencing | | OFT: Prebiotics normalized the duration spent in the center zone without influencing the traveled distances; FST: No observable effects were linked to prebiotics. | Distinct differences in microbiota composition were observed between female and male mice. |  |  |  |
|  | | |  | | | **Diets** | | |  | | | |  | **Diets** |
| Provensi et al., 2019^11^ | / | Male Wistar Rats | | enriched diet with the ω-3 polyunsaturated fatty acids eicosapentaenoic acid, docosahexaenoic acid, and docosapentaenoic acid and vitamin A (SED) | SPT | | EPM | / | | SPT: No observable impact on behavior resembling anhedonia; EPM: Dietary changes did not influence the frequency of entries or the duration spent in the center. | / |  |  |  |
| Takahashi et al., 2022^12^ | 80 | 3 weeks old C57BL/JJmsSLc (B6) mice | | Non-purified (CRF-1) and semi-purified (AIN-93G) commercial diets | FST; SPT | | / | 16S rRNA gene sequencing | | FST: AIN-93G was more effective in reducing the duration of immobility; SPT: Mice on the L-AIN diet displayed a lower sucrose preference compared to those on the L-CRF diet. | The *Firmicutes* to *Bacteroidetes* ratio and diversity was lower in the 5-week AIN-93G group than the 1-week AIN-93G group |  |  |  |
| Winther et al., 2015^13^ | 30 | 8 weeks male C57BL/6NBomTac mice | | Magnesium deficient diet (MgD) | FST | | OFT | 16S rRNA gene sequencing | | FST: mice fed with MgD showed higher immobility time than controls; OFT: There were no notable variations in the number of times entry was made into the center. | The gut microbiota composition of MgD mice varied considerably from mice on a standard control diet. |  |  |  |

*Note*. AFR = Animal Facility Reared; EPM = Elevated Plus Maze Test; FST = Fast Swim Test; MS = Maternal Separation; OFT = Open Field Test; Pro = Probiotic; SIT = Social Interaction Test; SPT = Sucrose Preference Test; TST = Tail Suspension Test

Table S2 **Details of the Search Terms**

| **Search Terms** | | |
| --- | --- | --- |
| Gut Microbiota-based Interventions | Microbiome and the MGB axis | microbiome/microbiota/ Brain-Gut Axis/ Gut-Brain-Axis/gut microbiota modulation/gut microbiome modulation).mp. |
|  | Microbiota interventions | (probiotic* or psychobiotics* or prebiotic* or postbiotics* or antibiotic* or diet* or faecal microbiota transplantation* * or synbiotics* or probiotic supplementary* or prebiotic supplementary* or probiotic formulas* or prebiotic formulas* or dietary*or paraprobiotics*or fermented foods* or dietary fiber* or dietary supplementary*).mp. |
|  | Depression and anxiety | (depression* or anxiety* or depressive* or anxious* or stress* or mental health* or psychiatric disorder* or neuropsychiatric disorder* or psychological* or Depressive Disorder* or mood disorder* or mental distress*).mp. |
|  | Children and adolescents | (child* or adolescent* or pediatric* or school-age or school children or teenager or youth or preadolescent or pre-teen or puberty or pubertal).mp. |

Table S3. PICOS criteria for inclusion and exclusion of studies

| Parameter | Inclusion Criteria | Exclusion Criteria |
| --- | --- | --- |
| Population | Human children and adolescents with depressive or anxiety symptoms measured by validated scales | Adult or infant populations, rodents/animals |
| Interventions | Gut microbiota-based interventions | Other interventions unrelated to gut microbiota (e.g., psychological, pharmacological therapies, SSRIs or psychotherapy) |
| Comparators Outcomes | No gut microbiota-based interventions, such as placebo or other interventions | Interventions without valid control or comparator groups. |
| Outcomes | Depression and anxiety, depressive- or anxiety-like symptoms, regardless of whether these are the primary outcomes; other mental health measures included (e.g., ASD and anorexia nervosa symptoms). | Studies not including measures of depressive or anxiety symptoms. |
| Study design | Experimental randomized controlled trials (RCTs) | reviews, brief reports, protocol, observational studies |

Abbreviations: ASD, Autism Spectrum Disorder

This systematic review was registered in the PROSPERO database on October 16, 2023 (CRD42023468511).

**Appendix S1**

An example of quality assessment using the Revised Cochrane risk-of-bias tool for randomized trials (RoB 2)

# Considerations for risk of bias

**Trial**: Amat-Bou et al 2020

**Comparison**: probiotic vs placebo

**Outcome**: the potential benefits of probiotic BPL1 on cardiometabolic risk factors in subjects with Prader-Willi syndrome.

**Result**: Rate reduction of 36% (95% confidence interval 4% to 57%)

## 1.Risk of bias arising from the randomization process

Comment on whether randomization was performed well, with the allocation sequence concealed until participants (or clusters) were enrolled and assigned to interventions. If not, could this have introduced bias in the result?

| *Answer:*   - 1. Y   2. Y   3. N |
| --- |

*Assign a category:*

| Low Concerns | MARK |
| --- | --- |
| Some Concerns |  |
| High Concerns |  |

## 2.Risk of bias due to deviations from the intended interventions

Comment on whether participants and trial personnel were aware of their intervention group during the trial. If not, could knowledge of the intervention have led these people to do things they would not have done outside of a trial? (and could these have impacted on participants’ outcomes, leading to bias?)

| *Answer:*  2.1 N  2.2 N  2.6 Y |
| --- |

*Assign a category:*

| Low Concerns | MARK |
| --- | --- |
| Some Concerns |  |
| High Concerns |  |

## 3.Risk of bias due to missing outcome data

Comment on the completeness of follow up. If participants did not contribute outcome data, might this introduce bias? (Could ‘missingness’ be related to the actual outcome participants had?)

| *Answer:*  3.1 Y |
| --- |

*Assign a category:*

| Low Concerns | MARK |
| --- | --- |
| Some Concerns |  |
| High Concerns |  |

## 4.Risk of bias in measurement of the outcome

Comment on whether outcome assessors were blinded to intervention group. If not, could knowledge of the intervention have affected the measurements they made?

| *Answer:*  4.1 N  4.2 N  4.3 NI  4.4 N |
| --- |

*Assign a category:*

| Low Concerns | MARK |
| --- | --- |
| Some Concerns |  |
| High Concerns |  |

5.Risk of bias in selection of the reported result

Comment on whether the result you are looking at could have been selected for publication because the finding was exciting (versus whether the authors were just following through what they said they would do in the trial protocol).

| *Answer:*  NCT03548480  5.1 Y  5.2 PN  5.3 N |
| --- |

*Assign a category:*

| Low Concerns | MARK |
| --- | --- |
| Some Concerns |  |
| High Concerns |  |

Overall risk of bias in the result

Comment on your overall level of concern about risk of bias in the result. Support your answer by drawing on your risk of bias assessments for each domain above

| *Answer:*  LOW RISK OF BIAS |
| --- |

1. Desbonnet L, Garrett L, Clarke G, Kiely B, Cryan JF, Dinan TG. Effects of the probiotic Bifidobacterium infantis in the maternal separation model of depression. *Neuroscience*. 2010;170(4):1179-1188. doi:10.1016/j.neuroscience.2010.08.005

2. Karen C, Shyu DJH, Rajan KE. <i>Lactobacillus paracasei</i> Supplementation Prevents Early Life Stress-Induced Anxiety and Depressive-Like Behavior in Maternal Separation Model-Possible Involvement of Microbiota-Gut-Brain Axis in Differential Regulation of MicroRNA124a/132 and Glutamate Receptors. *FRONTIERS IN NEUROSCIENCE*. AUG 31 2021;15719933. doi:10.3389/fnins.2021.719933

3. Kosuge A, Kunisawa K, Arai S, et al. Heat-sterilized Bifidobacterium breve prevents depression-like behavior and interleukin-1β expression in mice exposed to chronic social defeat stress. *Brain, behavior, and immunity*. 2021;96:200-211. doi:10.1016/j.bbi.2021.05.028

4. Liao JF, Hsu CC, Chou GT, Hsu JS, Liong MT, Tsai YC. Lactobacillus paracasei PS23 reduced early-life stress abnormalities in maternal separation mouse model. *Benef Microbes*. Apr 19 2019;10(4):425-436. doi:10.3920/bm2018.0077

5. Liu Y-W, Liong MT, Chung Y-CE, et al. Effects of lactobacillus plantarum PS128 on children with autism spectrum disorder in Taiwan: A randomized, double-blind, placebo-controlled trial. *Nutrients*. 2019;11(4):820. doi:10.3390/nu11040820

6. Maehata H, Kobayashi Y, Mitsuyama E, et al. Heat-killed Lactobacillus helveticus strain MCC1848 confers resilience to anxiety or depression-like symptoms caused by subchronic social defeat stress in mice. *Bioscience, biotechnology, and biochemistry*. 2019;83(7):1239-1247. doi:10.1080/09168451.2019.1591263

7. Wei C-L, Wang S, Yen J-T, et al. Antidepressant-like activities of live and heat-killed Lactobacillus paracasei PS23 in chronic corticosterone-treated mice and possible mechanisms. *Brain research*. 2019;1711:202-213. doi:10.1016/j.brainres.2019.01.025

8. Burokas A, Arboleya S, Moloney RD, et al. Targeting the microbiota-gut-brain axis: Prebiotics have anxiolytic and antidepressant-like effects and reverse the impact of chronic stress in mice. Psychopharmacology 2580. *Biological Psychiatry*. 2017;82(7):472-487. doi:<https://dx.doi.org/10.1016/j.biopsych.2016.12.031>

9. Chi X, Pan CQ, Liu S, Cheng D, Cao Z, Xing H. Regulating Intestinal Microbiota in the Prevention and Treatment of Alcohol-Related Liver Disease. *Canadian Journal of Gastroenterology and Hepatology*. 2020;2020:6629196. doi:<https://dx.doi.org/10.1155/2020/6629196>

10. Jiang J, Fu Y, Tang A, et al. Sex difference in prebiotics on gut and blood–brain barrier dysfunction underlying stress‐induced anxiety and depression. *CNS Neuroscience & Therapeutics*. 2023;

11. Provensi G, Schmidt SD, Boehme M, et al. Preventing adolescent stress-induced cognitive and microbiome changes by diet. *Proceedings of the National Academy of Sciences of the United States of America*. 2019;116(19):9644-9651. doi:<https://dx.doi.org/10.1073/pnas.1820832116>

12. Takahashi E, Ono E. Differential effects of different diets on depressive-like phenotypes in C57BL/JJmsSLc mice. Physiological Processes 2540. *Physiology & Behavior*. 2022;243doi:<https://dx.doi.org/10.1016/j.physbeh.2021.113623>

13. Winther G, Pyndt Jørgensen BM, Elfving B, et al. Dietary magnesium deficiency alters gut microbiota and leads to depressive-like behaviour. *Acta neuropsychiatrica*. 2015;27(3):168-176. doi:10.1017/neu.2015.7
